# VEGF Production by Ly6C^+high^ Monocytes Contributes to Ventilator-Induced Lung Injury

**DOI:** 10.1371/journal.pone.0165317

**Published:** 2016-10-26

**Authors:** Chung-Sheng Shi, Tzu-Hsiung Huang, Chin-Kuo Lin, Jhy-Ming Li, Mei-Hsin Chen, Mei-Ling Tsai, Chih-Ching Chang

**Affiliations:** 1 Graduate Institute of Clinical Medicine Sciences, College of Medicine, Chang Gung University, Taoyuan, Taiwan; 2 Division of Urology, Department of Surgery, Chang Gung Memorial Hospital, Chiayi, Taiwan; 3 Department of Respiratory Therapy, Chang Gung Memorial Hospital, Chiayi, Taiwan; 4 The Division of Pulmonary and Critical Care Medicine, Chang Gung Memorial Hospital, Chiayi, Taiwan; 5 Department of Physiology, Medical College, National Cheng Kung University, Tainan, Taiwan; 6 Department of Environmental and Occupational Health, Medical College, National Cheng Kung University, Tainan, Taiwan; Medical University of South Carolina, UNITED STATES

## Abstract

**Background:**

Mechanical ventilation is a life-saving procedure for patients with acute respiratory failure, although it may cause pulmonary vascular inflammation and leakage, leading to ventilator-induced lung injury (VILI). Ly6C^+high^ monocytes are involved in the pathogenesis of VILI. In this study, we investigated whether pulmonary infiltrated Ly6C^+high^ monocytes produce vascular endothelial growth factor (VEGF) and contribute to VILI.

**Methods:**

A clinically relevant two-hit mouse model of VILI, with intravenous lipopolysaccharide (LPS, 20 ng/mouse) immediately before high tidal volume (HTV, 20 mL/kg) ventilation (LPS+HTV), was established. Blood gas and respiratory mechanics were measured to ensure the development of VILI. Flow cytometry and histopathological analyses revealed pulmonary infiltration of leukocytes subsets. Clodronate liposomes were intravenously injected to deplete pulmonary monocytes. *In vitro* endothelial cell permeability assay with sorted Ly6C^+high^ monocytes condition media assessed the role of Ly6C^+high^ monocytes in vascular permeability.

**Results:**

LPS+HTV significantly increased total proteins, TNF-α, IL-6, vascular endothelial growth factor (VEGF) and mononuclear cells in the bronchoalveolar lavage fluid (BALF). Pulmonary Ly6C^+high^ monocytes (SSC^low^CD11b^+^F4/80^+^Ly6C^+high^), but not Ly6C^+low^ monocytes (SSC^low^CD11b^+^F4/80^+^Ly6C^+low^), were significantly elevated starting at 4 hr. Clodronate liposomes were able to significantly reduce pulmonary Ly6C^+high^ monocytes, and VEGF and total protein in BALF, and restore PaO_2_/FiO_2_. There was a strong correlation between pulmonary Ly6C^+high^ monocytes and BALF VEGF (*R*^2^ = 0.8791, *p*<0.001). Moreover, sorted Ly6C^+high^ monocytes were able to produce VEGF, resulting in an increased permeability of endothelial cell monolayer in an *in vitro* endothelial cell permeability assay.

**Conclusion:**

VEGF produced by pulmonary infiltrated Ly6C^+high^ monocytes regulates vasculature permeability in a two-hit model of HTV-induced lung injury. Ly6C^+high^ monocytes play an important role in the pathogenesis of VILI.

## Introduction

Mechanical ventilation is an important life-saving procedure, but often causes lung injury due to regional over-distension of pulmonary alveoli, particularly with larger tidal volumes, and leads to ventilator-induced lung injury (VILI) [1, 2]. VILI is characterized by increased recruitment of leukocytes that mediate acute inflammatory responses by releasing inflammatory chemokines, cytokines, and mediators. These molecules increase the pulmonary-vasculature permeability and leakage, resulting in protein-rich pulmonary edema that ultimately impairs gas exchange [3, 4].

Alveolar macrophages and neutrophils, with the release of inflammatory mediators, are critically involved in the pathogenesis of lung injury [5, 6]. Pulmonary margination of monocytes also plays a significant role in LPS-induced system inflammation [7, 8]. Lung marginated monocytes are responsible for TNF-mediated microvascular endothelial cell activation and vascular injury in a subclinical low dose LPS-induced early endotoxemia [9]. Subclinical LPS challenge causes pulmonary margination of Gr-1^+high^ monocytes within 2 hr, and this greatly enhances the development of sepsis-related increases in pulmonary vascular leakage [9, 10]. Lung marginated Gr-1 (Ly6C/G) monocytes are involved in the progression of VILI, in a two-hit model with a low dose of LPS 2 hr before high stretch ventilation (V_T_ 34–36 mL/kg), and contribute to a decrease in lung compliance [11, 12]. However, further investigations are required with regard to the precise mechanism that Ly6C^+high^ monocytes play in the development of VILI.

VEGF is an endothelial cell-specific growth factor, and is involved in endothelial progenitor cells-dependent vasculogenesis and capillary endothelial cells-dependent angiogenesis [13, 14]. VEGF activates VEGF receptor-2 (VEGFR-2/Flk-1/KDR) and increases capillary permeability by tyrosine phosphorylation of the interendothelial adhesion molecule VE cadherin or through enhancing the production of nitric oxide and prostacyclin and increasing vascular permeability [15, 16]. VEGF mRNA expression is associated with neutrophils influx and increased total protein in the BALF after exposure to LPS in mice [17]. In response to reactive oxygen species, lung stretch and inflammatory cytokines, VEGF can be produced from pulmonary type II cells, alveolar macrophages, and neutrophils [18, 19]. Serum VEGF is significantly increased in ventilation-induced lung injury, and implicated in mediating endothelial NOS induced systemic microvascular leakage [20]. VEGF siRNA is shown to reduce high stretch ventilation (30 mL/Kg, 65 breaths/min) induced VEGF production, protein leakage, and lung injury score [21].

In this study, we hypothesized that pulmonary infiltrated Ly6C^+high^ monocytes contribute to the increase in VEGF production and are responsible for the development of VILI. We explored the kinetic changes of pulmonary vascular leakage, Ly6C^+high^ monocytes influx, and cytokine and VEGF production in a clinically relevant two-hit mouse model of VILI. *In vivo* depletion of Ly6C^+high^ monocytes and *in vitro* endothelial cell permeability assay using condition media were applied to strengthen their role during VILI.

## Methods

### Animals

Male C57BL/6 mice between 6 and 8 weeks of age and weighing 20–25 g were obtained from the National Laboratory Animal Center (Taipei, Taiwan). All animal experiments were conducted according to the NIH guidelines (Guide for the Care and Use of Experimental Animals). The procedures were approved by the Institutional Animal Care and Use Committee of Chang Gung Memorial Hospital (Chiayi, Taiwan).

### Experimental models of mechanical ventilation

We modified a two-hit protocol with an intravenous subclinical low dose (20 ng/mouse) of LPS (O111B4; Sigma-Aldrich, St Louis, MO, USA) immediately before HTV ventilation (VT 20 mL/Kg, 60 breaths/min) or LTV ventilation (VT 7 mL/Kg, 90 breaths/min). Briefly, a 20-gauge angiocatheter was introduced into the tracheotomy orifice of mice under general anesthesia using intraperitoneal Zoletil 50 (80 mg/kg; Tiletamine-Zolazepam, Virbac, Carros CEDEX, France), anesthesia was sustained with Zoletil 50 (10 mg/Kg/h) during mechanical ventilation. The mice were placed in a supine position on a heating blanket and then attached to a specialized rodent ventilator (SAR-830/AP; CWE Inc., Ardmore, PA, USA) and received HTV or LTV while breathing room air with zero end-expiratory pressure. LTV was applied in this study to mimic the clinical setting of mechanical ventilation. A moderate HTV (20 mL/Kg) was applied to mimic heterogeneous over-distension of the injured lung, resulting from partially collapsed or fluid-filled lungs [22, 23]. Respiratory mechanics were measured from end-inspiratory occlusions after constant flow inflation [24–26]. Mice were euthanized using an overdose of anesthetic at 0, 2, 4, 6, and 8 hr. Non-ventilated mice served as the controls.

To investigate the role of Ly6C^+^ monocytes in the pathogenesis of VILI, intravenous clodronate liposome (200 μL/mouse; Clodronateliposomes.com, Haarlem, the Netherlands) was applied 24 hr before the start of ventilation to deplete monocytes.

### Blood collection, bronchoalveolar lavage, analysis of BAL leukocytes

The blood samples of the mice were obtained by cardiac puncture for the measurement of TNF-α, IL-6, and VEGF, and analysis of blood gas was carried out using a gas analyzer to reflect pulmonary oxygenation and the severity of acute lung injury. The lung was exposed, and the trachea was cannulated for lavaging the bronchoalveolar (BAL) space three times with 1 mL phosphate-buffered saline (PBS). BAL fluid (BALF) was centrifuged at 300 *× g* for 10 min, and the cell pellets were re-suspended in 1 mL RPMI1640 medium. An aliquot of the cell suspension was used to examine total cell numbers by a hemocytometer (Paul Marienfeld GmbH, Lauda-Koenigshofen, Germany). The primary supernatant was used to measure the level of total protein, TNF-α, IL-6, and VEGF. In addition, 3 x 10^4^ cells were centrifuged at 180 g for 5 min onto glass slides to prepare cytospin slides (Thermo Electron Corporation, Waltham, MA, USA). Following Liu staining, differential counts of neutrophils and mononuclear cells were determined by counting 400 cells under a microscope (Magnification x 400: Olympus, Tokyo, Japan).

### Quantitation of total protein, TNF-α, IL-6, and VEGF in BALF

BALF total protein was measured using a Pierce protein assay kit, according to the manufacturer’s manual (Pierce, Rockford, IL, USA). Enzyme-linked immunosorbent assay (ELISA) was adopted to measure the mouse TNF-α, IL-6, and VEGF, according to the manufacturer’s protocols (R&D systems, Chantilly, VA, USA). VEGF ELISA detects mature VEGF containing 120 and 164 amino acid residues (secreted VEGF).

### Flow cytometric analysis

Following sacrifice, the lungs were removed, homogenized finely, and incubated in Digest buffer (RPMI1640 media with Dispase II [2.5 U/mL] and DNase I [30 μg/mL]) for 45 min at 37°C. The cells were then filtered through a 100 μm cell strainer (BD Biosciences, San Jose, CA, USA) and 41 μm nylon filter mesh to obtain single cell suspensions.

To characterize mouse lung neutrophils and monocytes, the cells were labelled using fluorophore-conjugated anti-mouse antibodies (eBioscence, San Diego, CA, USA) against CD11b (1:200), Ly6C (Clone HK1.4, specifically recognizing the Ly6C epitope; 1:200), F4/80 (1:300), or an appropriate isotype-matched control. Cells were analyzed using a FACSCanto II with FACSDiva software (BD Biosciences) and FlowJo software (Tree Star, Ashland, OR) to identify and quantify neutrophils, and Ly6C^+high^ and Ly6C^+low^ monocyte subsets. In addition, the viable monocytes with CD11b, F4/80, and Ly6C positive staining were sorted using the FACSAria Fusion cell sorter (BD Biosciences) for the following *ex vivo* VEGF production or *in vitro* endothelial cell permeability assay.

Cells with high side scatter (SSC) signal and CD11b expression were gated (G4), and the resulting cell population was further analyzed for F4/80 and Ly6C expression. Neutrophils were identified as having high levels of SSC signal and CD11b expression, being negative for F4/80 and having intermediate high level of Ly6C (G5, SSC^high^CD11b^+high^F4/80^-^Ly6C^+inter^). Those low SSC and CD11b-positive events (G1) were gated for further analysis of Ly6C and F4/80 expression. Cells in G2 were positive for F4/80 and had high Ly6C expression, and were recognized as Ly6C^+high^ monocytes (SSC^low^CD11b^+^F4/80^+^Ly6C^+high^). Those in G3 were SSC^low^CD11b^+^F4/80^+^Ly6C^+low^ cells (Ly6C^+low^ monocytes).

To analyze VEGF expression, the cells were permeabilized using Cytofix/Cytoperm solution and Perm/Wash Buffer (BD Biosciences), and stained with anti-VEGF antibodies (1:200, Santa Cruz Biotechnology, Santa Cruz, CA, USA; PE-conjugated secondary Ab, 1:300, Santa Cruz), in addition to CD11b and Ly6C, and analyzed using a FACSCanto II.

### Histopathological evaluation

After the mice were sacrificed, the lungs were fixed by instillation of 10% formaldehyde, and the specimens were then immerged in 4% paraformaldehyde at 4°C for 48 hr. After fixation and washing, the specimens were embedded in paraffin wax. A 5 μm thick section from each paraffin block was expanded on slide and stained with hematoxylin and eosin (H&E; Vector Labs, Burlingame, CA, USA). The histopathologic analysis of lung injury was performed by examining the recruitment of leukocytes on a microscope at 400× magnification.

### *Ex vivo* VEGF production assay

Sorted Ly6C^+high^ monocytes from LPS+HTV injured lungs were seeded (1×10^5^ cells per well) in a 96-well plate and cultured in RPMI medium containing 10% fetal bovine serum. At 0, 1, 6, and 12 hr, Ly6C^+high^ monocytes conditioned medium (CM) was collected for detecting the secretion of VEGF, using ELISA (R&D Systems), according to the manufacturer’s instructions. Ly6C^+high^ monocytes CM from 6 hr culture were used for the following *in vitro* endothelial permeability assay.

### *In vitro* endothelial cell permeability assay

To evaluate the effect of Ly6C^+high^ monocytes on the permeability of endothelial cells, we measured the flux of streptavidin-conjugated horseradish peroxidase (streptavidin-HRP; R&D systems) across the endothelial cell-coated monolayer on Transwell filters (0.4 μm pore polycarbonate filters; Corning Costar Co., Cambridge, MA, USA) [27]. Briefly, 100 μL of mouse ECs (1×10^5^ cells, SVEC4-10; Bioresource Collection and Research Center, BCRC, Hsinchu, Taiwan), which is an axillary lymph node vessel EC cell line with SV40 transformation and cultured in Dulbecco's modified Eagle's medium containing 10% heat-inactivated fetal bovine serum, were added to the upper well for 24–48 hr. The lower wells were filled with 600 μL of medium to support cell growth. After reaching confluence, ECs were washed and treated with Ly6C^+high^ monocyte conditioned media, collected from a 6-hr culture, in the presence or absence of anti-VEGF antibody (20 μg/mL; BioLegend, San Diego, CA, USA), or isotype-matched rat IgG2a control (20 μg/mL). After 6 hr incubation, streptavidin-HRP (1: 200 dilution) was added to the upper wells and incubated at 37°C for 30 min, and the medium in the lower wells was collected for assaying HRP activity using 3,3',5,5'-tetramethylbenzidine, according to the manufacturer’s instructions (Sigma-Aldrich). The signals emitted by streptavidin-HRP were expressed as a percentage of the untreated control.

### Statistical analysis

Data are expressed as means ± standard deviation (SD). Statistical comparisons were made using unpaired Student’s t-tests for end point data. For some end point analyses, data were compared with those of non-ventilated mice for qualitative purposes. Comparisons among multiple groups were made by one-way analysis of variance with Bonferroni-corrected pairwise post hoc comparisons (Prism software, version 5.0). The relationship between VEGF production and total proteins, and Ly6C^+high^ monocyte and VEGF production was evaluated using the coefficient of determination (R^2^) in the goodness of fit model. Statistical significance was defined as *P* <0.05.

## Results

### Two-hit model of VILI, LPS with HTV ventilation

**Fig 1:**
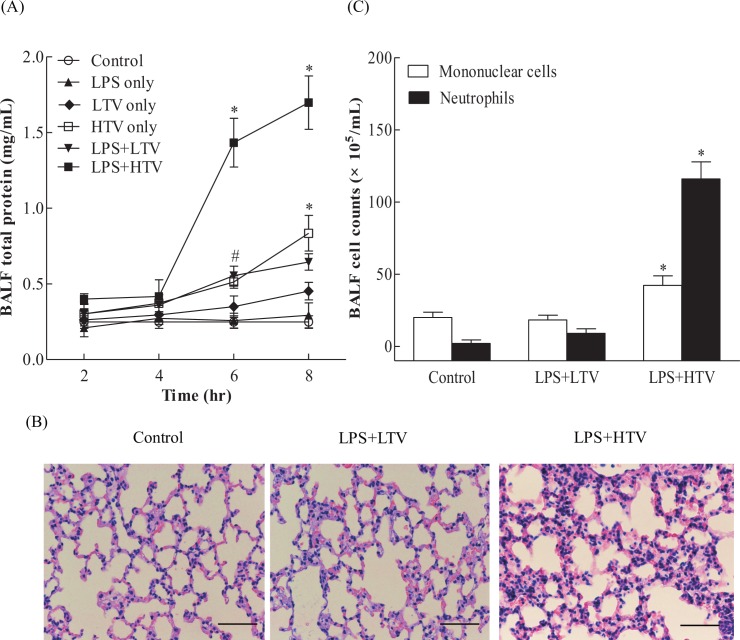
LPS+HTV significantly promoted the increase of pulmonary-vasculature leakage and leukocyte recruitment. (A) Time-dependent changes of BALF total protein, (B) histopathology (H&E stain) at 6 hr, and (C) differential cell counts at 6 hr. Scale bar = 200 μm. Values represent the mean ± SD (*n* = 4–6). ^#^*p* <0.05 and *p <0.01, compared with the control at that time point.

Total proteins in the BALF were measured as an indicator of alveolar-capillary permeability. In one-hit models, LPS or LTV ventilation alone did not induce a significant increase in total protein in the BALF, indicating that they did not cause any vascular leakage. However, in mice that received HTV ventilation as the only treatment, the BALF total proteins significantly increased starting at 6 hr, and similar profiles in protein leakages could be found in the mice that received LPS plus LTV ventilation (LPS+LTV). LPS plus HTV ventilation (LPS+HTV) started to cause a significant increase in protein leakage at 4 hr, and the levels of total proteins at 6 and 8 hr were significantly higher than those in HTV alone or LPS+LTV group (Fig 1A).

At 6 hr, a histopathological examination revealed that thickened alveolar walls and leukocytes infiltration were found in the lungs of mice that received LPS+HTV. There were less obvious changes in the mice lungs that received LPS+LTV. The alveolar walls remained intact in non-ventilated control mice (Fig 1B).

Fig 1C shows that BALF neutrophils and mononuclear cells were significantly increased in LPS+HTV treated mice at 6 hr, compared with non-ventilated control mice. There were no significant changes in mononuclear cells and neutrophils in mice challenged with LPS+LTV, compared to the results seen with the control mice. Therefore, BAL leukocytes with differential counts and the results of the histopathological study revealed the infiltration of inflammatory cells in the alveolar spaces and thickened alveolar walls.

As shown in Table 1, plateau inspiratory pressure (Pplat) at 6 hr reached 20.8±1.84 cmH_2_O, about a 35% increase from baseline at 15.4±0.48 cmH_2_O. This supports the occurrence of substantial pulmonary edema at 6 hr post-exposure. Moreover, respiratory system compliance (Crs) and pulmonary oxygenation (PaO2/FiO2) were deteriorated in LPS+HTV treated mice, compared with those of non-ventilated control mice or LPS+LTV treated mice. No significant differences in pH, PaCO_2_, or HCO_3_ were found among the four experimental groups (Table 1), indicating that there was no severe acidosis during the experiments.

**Table 1:** Pathophysiological variables in ventilated mice, with or without clodronate liposome pretreatment.

Group	A. Control	B. LPS+LTV	C. LPS+HTV	D. LPS+HTV +200 μL Clod	D *v*.*s*. C
**Blood gas analysis**					
PH	7.43±0.028	7.41 ±0.01	7.32 ±0.022	7.36 ±0.024	NS
PaCO_2_ (mmHg)	37.7±2.06	35.2 ±1.25	36.1±1.94	36.7±1.70	NS
HCO_3_ (mmol/L)	24.0 ±0.81	22.2±0.5	18.0 ±0.89	20.2 ±0.95	NS
PaO_2_ (mmHg)	90.2±4.11	80.5±3.69^**#**^	53.6±5.08*	69.5±4.20^**#**^	*p* <0.01
PaO_2_/FiO_2_ ratio	429.0±19.6	383.2±17.5^**#**^	255.5±23.9*	330.5±20.2^**#**^	*p* <0.01
**Respiratory mechanics**					
PIP (cmH_2_O) at start	ND	8.0±0.81	17.0±0.89	16.8±0.96	NS
PIP (cmH_2_O) at 6 hr	ND	10.2±0.95	24.3±0.81^**†**^	20.7±0.95^**†**^	NS
Pplat (cmH_2_O) at start	ND	6.55±0.34	15.4±0.48	15.9±0.62	NS
Pplat (cmH_2_O) at 6 hr	ND	7.18±0.3	20.8±1.84^**†**^	18.3±0.46^**†**^	NS
Crs (mL/kg/cmH_2_O) at start	ND	1.22±0.06	1.3±0.04	1.26±0.05	NS
Crs (mL/kg/cmH_2_O) at 6 hr	ND	1.11±0.05	0.97±0.08	1.09±0.03	NS
ΔCrs (%)	ND	-9.2±0.8	-25.5±5.5	-12.0±2.18	*p* <0.01

Respiratory mechanics (PIP and Pplat) were measured by occluding the airway at the end of inspiration. Change in respiratory mechanics was defined as the percentage increase or decrease in Crs [ΔCrs (%), Crs (mL/kg/cmH_2_O) = TV/ Pplat] at 6 hr versus the value at start. LPS, lipopolysaccharide; LTV, low tidal volume ventilation; HTV, high tidal volume ventilation; Clod, clodronate liposomes; Crs, respiratory system compliance; PIP, peak inspiratory pressure; Pplat, plateau inspiratory pressure; TV, tidal volume; ND, not determined; NS, not significant. Values represent the mean ± SD (*n* = 4–6).

^#^*p* <0.05 and

*p <0.01, compared with the control.

^†^ *p* <0.01, compared with the value at start.

### Production of TNF-α, IL-6, and VEGF in VILI

**Fig 2:**
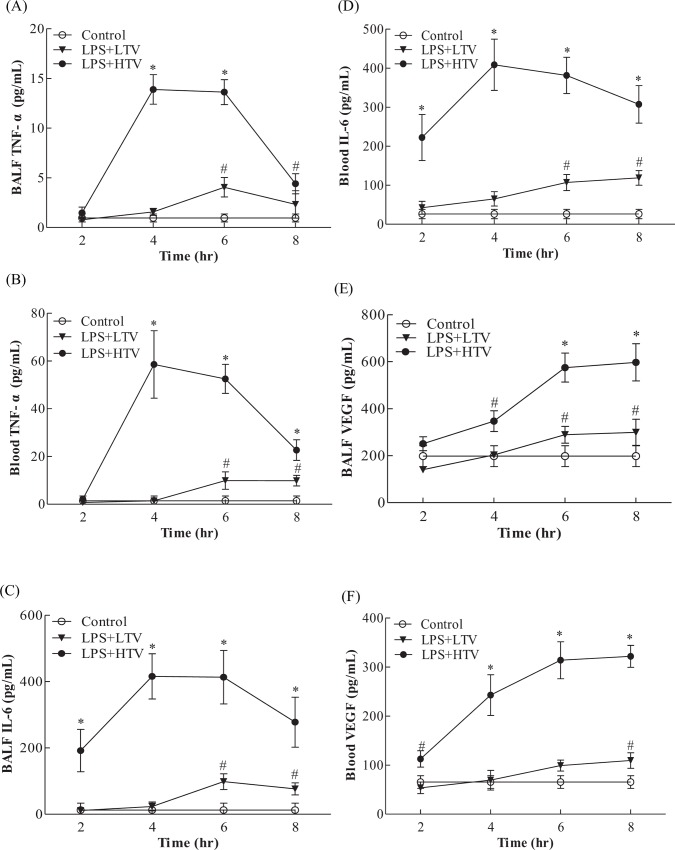
**Time dependent effects of LPS+HTV on** (A) BALF TNF-α, (B) blood TNF-α, (C) BALF IL-6, (D) blood IL-6, (E) BALF VEGF and (F) blood VEGF. Values represent the mean ± SD (*n* = 4–6). ^#^*p* <0.05 and *p <0.01, compared with the control at that time point.

In LPS+HTV treated mice, TNF-*α* and IL-6 in the BALF and blood reached the maximal level at 4 hr, and this was maintained at similar levels at 6 hr, both of which were significantly higher than those seen in the control or LPS+LTV mice (Fig 2A–2D). When compared with control or LPS+LTV, LPS+HTV caused significant increases of BALF VEGF at 4, 6 and 8 hr. BALF VEGF reached the highest level at 6 hr, and was at a similar level at 8 hr. Blood VEGF was found to be significantly elevated starting at 2 hr, reached the highest levels at 6 and 8 hr (Fig 2E and 2F).

### The recruitment of Ly6C^+high^ monocytes and neutrophils

**Fig 3:**
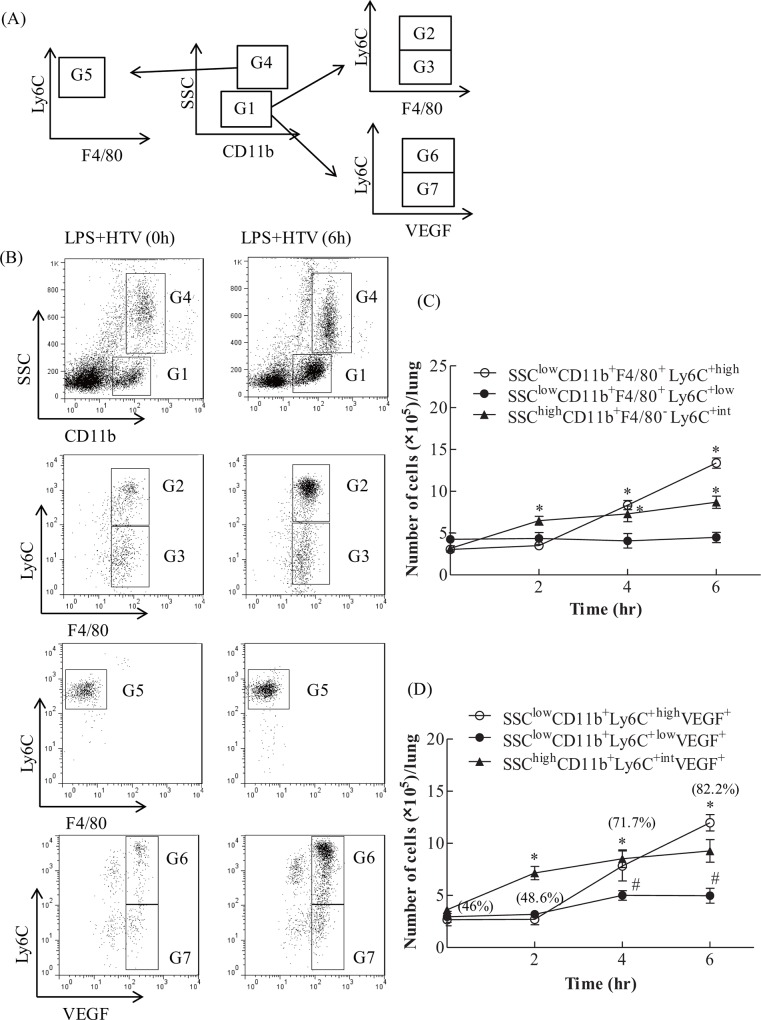
Ly6C^+high^, but not Ly6C^+low^, monocytes and neutrophils were recruited into ventilator-injured lungs. (A) Gating strategy for differentiating Ly6C monocyte subsets and neutrophils. (B) Low SSC and CD11b-positive population (G1) was gated for further analysis of Ly6C and F4/80 or VEGF expressions. High Ly6C and F4/80 positive cells (G2) represented Ly6C^+high^ monocytes. Low Ly6C and F4/80 positive cells (G3) represented Ly6C^+low^ monocytes. The high SSC and CD11b-positive population (G4) represented neutrophils. High SSC and CD11b-positive population (G4) was gated for further analysis of Ly6C and F4/80 expressions. Intermediate high Ly6C and F4/80 negative cells (G5) represented neutrophils. Further, low SSC and CD11b positive population (G1) was gated to analyze the intracellular expression of VEGF in Ly6C monocytes. High Ly6C and VEGF-positive (G6) cells represented the VEGF expressing-Ly6C^+high^ monocytes. Low Ly6C and VEGF-positive (G7) cells represented the VEGF expressing-Ly6C^+low^ monocytes. (C) Time course for the recruitment of Ly6C^+high^, Ly6C^+low^ monocytes, and neutrophils in the two-hit model of VILI. (D) Time course for the recruitment of VEGF expressing SSC^low^CD11b^+^Ly6C^+high^ or Ly6C^+low^ cells in the two-hit model of VILI. (Parenthesis): % of SSC^low^CD11b^+^Ly6C^+high^ cells positive for VEGF. Values represent the mean ± SD (*n* = 4–6). ^#^*p* <0.05 and *p <0.01, compared with the control of each time point.

The gating strategy for the flow cytometry analyses of stained cells are shown in Fig 3A and 3B, and outlined in the previous section. Ly6C^+high^ monocytes were identified as SSC^low^CD11b^+^F4/80^+^Ly6C^+high^ cells, Ly6C^+low^ monocytes as SSC^low^CD11b^+^F4/80^+^Ly6C^+low^ cells, and neutrophils as SSC^high^CD11b^+high^F4/80^-^Ly6C^+inter^ cells.

As shown in Fig 3C, in response to LPS+HTV, pulmonary neutrophils were significantly elevated at 2 hr and maintained at similar levels throughout the study period, when compared with those at 0 hr. Pulmonary Ly6C^+high^ monocytes started to be significantly increased at 4 hr, with further elevation at 6 hr. However, there were no significant changes in the number of Ly6C^+low^ monocytes throughout the study period.

Similarly, SSC^low^CD11b^+^Ly6C^+high^VEGF^+^ cells were significantly increased at 4 h, compared with those at 0 hr, and reached a much higher level at 6 hr. SSC^low^CD11b^+^Ly6C^+high^VEGF^+^ cells accounted for 82.2±2.72% of SSC^low^CD11b^+^Ly6C^+high^ cells at 6 hr, 71.7±1.42% at 4 hr, 48±5.35% at 2 hr, and 46.0±4.85% at 0 hr (Fig 3D). The data suggest that Ly6C^+high^ monocytes might serve as a source of VEGF following LPS+HTV exposure. In addition, the percentage of neutrophils expressing VEGF is 56.8±3.97% at 0 hr, 79.3±2.51% at 2 hr, 82.5±2.62% at 4 hr, and 84.8±2.56% at 6 hr.

### The involvement of Ly6C^+high^ monocytes in VILI

**Fig 4:**
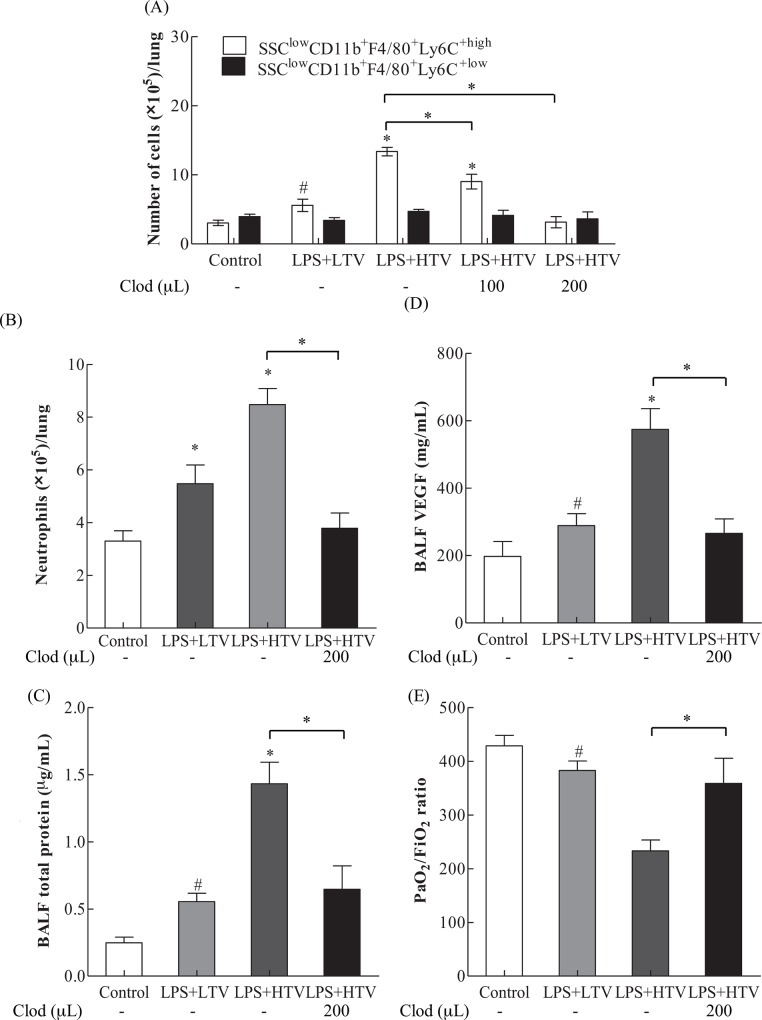
**Effects of Clodronate liposomes (200 μL/mice; 24 hr before LPS+HTV) on** (A) Ly6C^+high^ and Ly6C^+low^ monocytes, (B) neutrophils, (C) BALF total proteins, (D) BALF VEGF, and (E) PiO_2_/FiO_2_ ratio. Values represent the mean ± SD (*n* = 4–6). ^#^*p* <0.05 and *p <0.01, compared with the control or between groups.

To investigate the role of Ly6C^+high^ monocytes in the development of VILI, clodronate liposomes were used to deplete pulmonary leukocytes. The results from flow cytometry analysis shown in Fig 4A reveal that LPS+HTV treatment triggered a significant increase in Ly6C^+high^ monocytes. Pretreatment with clodronate liposomes for 24 hr significantly and dose-dependently reduced pulmonary infiltration of Ly6C^+high^ monocytes in LPS+HTV treated mice. However, clodronate liposome pretreatment had no effect on the pulmonary distribution of Ly6C^+low^ monocytes in LPS+HTV treated mice.

In addition, the number of pulmonary neutrophils was also significantly reduced by clodronate liposome pretreatment, as shown in Fig 4B.

Importantly, clodronate liposome pretreatment significantly prevented LPS+HTV-induced increases in BALF total proteins and VEGF (Fig 4C and 4D), and significantly reversed the LPS+HTV-induced reduction in PaO_2_/FiO_2_ ratio (Fig 4E, Table 1). These findings indicate that an improvement in the oxygenation index ensues following the reductions in vasculature leakages and edema.

**Fig 5:**
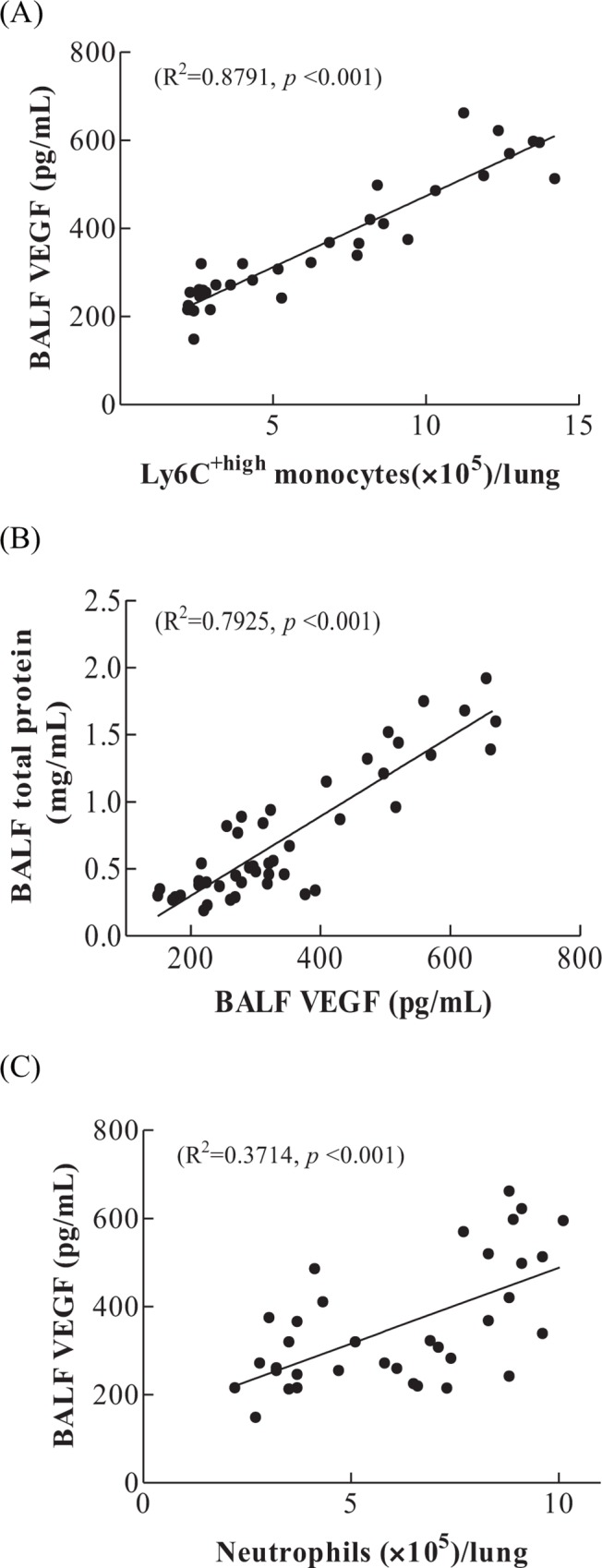
**Regression analysis** (A) Correlation of Ly6C^+high^ monocytes with BALF VEGF. Regression analysis, significant correlation between Ly6C^+high^ monocytes and BALF VEGF (*R*^2^ = 0.8791, *p*<0.001). (B) Correlation between VEGF with total proteins. Regression analysis, significant correlation between VEGF and total proteins (*R*^2^ = 0.7925, *p*< 0.001). (C) Correlation of neutrophils with BALF VEGF. Regression analysis, *R*^*2*^ = 0.3714, *p*<0.001.

To further explore the role of Ly6C^+high^ monocytes in LPS+HTV induced protein leakage, we first plotted the BALF VEGF against Ly6C^+high^ monocyte numbers, using data from Figs 2E, 3C, 4A and 4D. Analysis revealed a coefficient of determination (*R*^2^) of 0.8791 (*p*<0.001; Fig 5A). Furthermore, there was a strong correlation between BALF VEGF and total proteins (*R*^2^ = 0.7925, *p*< 0.001; Fig 5B), using data from Figs 1A, 2E, 4C and 4D.

Using data from Figs 2E, 3C, 4B and 4D, a coefficient of determination (*R*^*2*^) of 0.3714 (*p*<0.001) was found for the association between neutrophils and BALF VEGF (Fig 5C).

Taken together, the data strongly support that Ly6C^+high^ monocytes play a significant role in the VEGF production and increased alveolar-capillary permeability.

### *In vitro* endothelial cell permeability assay, VEGF-dependent mechanism

**Fig 6:**
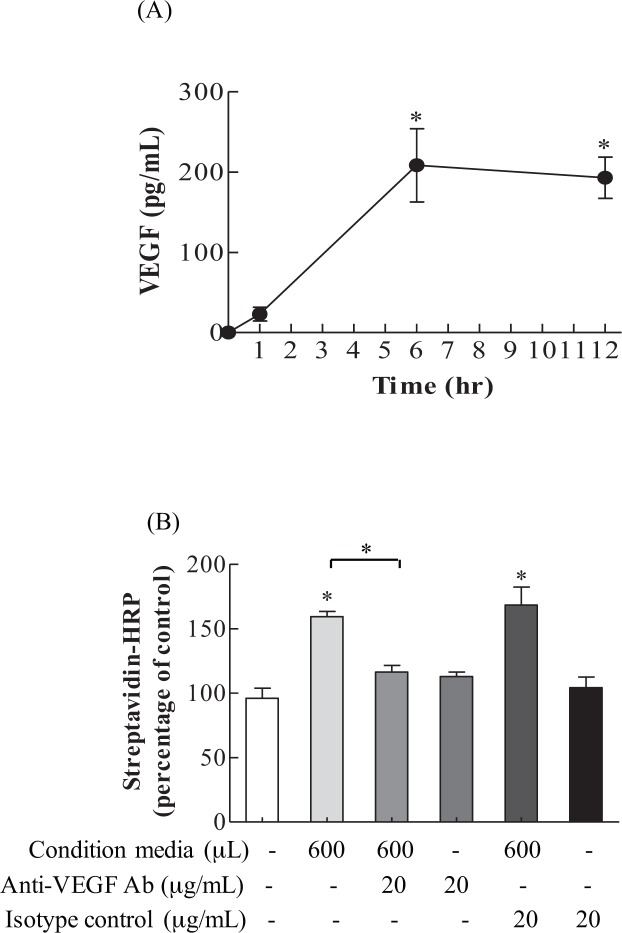
*In vitro* endothelial cell permeability, using condition media from *ex vivo* sorted Ly6C^+high^ monocytes culture. (A) VEGF production was significantly increased in sorted Ly6C^+high^ monocytes at 6 and 12 hr. Values represent the mean ± SD (*n* = 4). *p <0.001, compared with the control media at 0 hr. (B) Ly6C^+high^ monocytes conditioned media harvested from a 6-hr culture increased endothelial cell permeability, as indicated by the increased streptavidin-HRP in the lower chamber. Values represent the mean ± SD (*n* = 4). ^#^*p* <0.05 and *p <0.01, compared with the control or between groups.

To strengthen the hypothesis that Ly6C^+high^ and its VEGF play a significant role in pulmonary endothelial cell permeability, *in vitro* endothelial cell permeability assay was conducted with sorted Ly6C^+high^ monocyte conditioned media and anti-VEGF antibody. As shown in Fig 6A, sorted Ly6C^+high^ monocytes in culture produced a significant amount of VEGF at 6 and 12 hr. We next investigated whether Ly6C^+high^ monocyte conditioned media harvested at 6 hr caused a significant increase in the permeability of the endothelial cell monolayer. The results in Fig 6B demonstrate that, when compared with controls, the Ly6C^+high^ monocyte conditioned media caused significant increases in Streptavidin-HRP in the lower chamber, indicating increases in endothelial cell monolayer permeability. This effect was specifically inhibited by the addition of anti-VEGF antibody. Taken together, the results show that sorted Ly6C^+high^ monocytes are capable of secreting VEGF, leading to increased permeability in endothelial cells.

## Discussion

Patients with pre-existing lung infections often require ventilation support, leading to increased susceptibility to VILI, even with the use of protective ventilation measures [28, 29]. Subclinical endotoxemia via systemic LPS administration causes pulmonary infiltration of Ly6C^+high^ monocytes, and this increases the susceptibility of the lung to the development of acute lung injury [10]. In our experiment, intravenous low-dose LPS administration does not cause increased alveolar capillary permeability. HTV only results in less extent of alveolar capillary permeability, compared with two-hit model of intravenous LPS plus HTV, which aggravates the alveolar capillary permeability (Fig 1A). It is significant to note that Ly6C^+high^ monocyte infiltration and VEGF production are highly correlated in this study (Fig 5A). These Ly6C^+high^ monocytes are inflammatory monocytes expressing high levels of Ly6C and C-C chemokine receptor 2, and a low level of CX3C chemokine receptor 1 (Ly6C^+high^CCR2^+high^CX3CR1^+low^ monocytes) [30–32]. They are derived from bone marrow macrophage-dendritic precursor cells, and migrate to inflammatory loci. However, Ly6C^+low^CCR2^+low^CX3CR1^+high^ monocytes are resident monocytes, and migrate to tissues under resting or local inflammatory conditions. Thus, while Ly6C^+low^ monocytes remain constant throughout the study period, lung infiltrated Ly6C^+high^ monocytes increased significantly starting at 4 hr, which coincides with significant elevations in BALF total protein (Fig 1A).

Under normal conditions, VEGF is produced mainly by pulmonary epithelial cells and vascular smooth muscle. The alveolar space thus has a much higher VEGF level than pulmonary vasculature. However, in lung injury, including ventilator-induced lung injury, alveolar macrophages, monocytes and neutrophils become the major source of VEGF [19]. The injured alveolar epithelium enables the surged VEGF to gain easy access to the pulmonary vasculature and endothelial cells, resulting in increased vascular permeability and the subsequent elevated serum VEGF concentration [20]. In this work, blood VEGF is significantly increased starting at 2 hr (Fig 2F) and, importantly, pulmonary Ly6C^+high^ monocytes are significantly correlated with the production of BALF VEGF (Fig 5A), suggesting a switch from epithelial origin to myeloid cells in VEGF production. VEGF is capable of inducing the expression of adhesion molecules that bind leukocytes to endothelial cells [25, 30]. Leukocyte adhesion to endothelial cells triggers disorganization of endothelial cell adherens and tight junctions, and increases in vascular permeability. This is an important step proceeding leukocyte infiltration [31, 32]. Moreover, subclinical low dose LPS-induced endotoxemia primes Ly6C monocytes to develop enhanced proinflammatory capacities. These Ly6C monocytes possess increased cell-associated TNF signaling in pulmonary microcirculation, responsible for pulmonary endothelial cell activation and vascular injury [9, 10]. Therefore, in addition to VEGF production, Ly6C^+high^ monocytes may exert local cell-mediated effects on pulmonary microvasculature.

Redox imbalance occurs in ventilator-induced lung injury. Antioxidant Nrf transcription factor deficiency increases the susceptibility to VILI in mice [33]. N-acetylcysteine treatment attenuates ventilator-induced decreases in glutathione, inflammatory response and cell apoptosis [34]. Amifostine, a direct scavenger of reactive oxygen and nitrogen species, confers protection against ventilator-induced lung injury [35]. These findings suggest that oxidative stress plays an important role in the pathogenesis of VILI. Oxidative stress has been shown to increase VEGF production by activating VEGF promotor, located between -449 and -1, in macrophages [36], or through enhancing the binding of Sp1 and Sp3 transcription factors to proximal GC-rich motifs at -73/-66 and -58/-52 in gastric adenocarcinoma cells [37]. As such, Ly6C^+high^ monocytes may produce VEGF in response to ventilation-induced oxidative stress.

Neutrophils have been shown to play an important role in lung injury and VILI [5, 38]. The number of neutrophils is higher than that of Ly6C^+high^ monocytes in response to 2 hr high stretch ventilation in a two-hit model of VILI [11]. Similarly, we found that neutrophils outnumber Ly6C^+high^ monocytes at 2 hr (Fig 3C) and TNF-α is significantly increased starting at 4 hr post-exposure (Fig 2A and 2B). Neutrophils may serve as a source of TNF-α and VEGF in acute inflammatory response [39, 40]. TNF-α is able to alter the tight junction network, leading to increased permeability [41, 42]. The increased protein leakage may thus be in part caused by the increased TNF-α. At 6 hr, the number of Ly6C^+high^ monocytes is greater than that of neutrophils, which maintain constant numbers from 2 to 6 hr (Fig 3C). Research has shown that pulmonary Ly6C^+high^ monocytes may play a role in sustaining neutrophil influx in LPS-induced lung injury [43]. Pre-treatment with clodronate liposome was found to not affect neutrophils influx in non-ventilated lungs [11]. However, clodronate liposome-mediated depletion of Ly6C^+high^ monocytes attenuates the recruitment of neutrophils in the current study (Fig 4B). These findings suggest that decreased neutrophils may result from the clodronate liposomes-reduced Ly6C^+high^ monocytes, VEGF production and alveolar-capillary permeability. Our data thus provide further evidence for the significant role of Ly6C^+high^ monocytes in the development of VILI.

In normal skin tissue repair, inflammatory CCR2^+^Ly6C^+^ macrophages are most abundant during early wound repair, with only a small fraction of them expressing VEGF. However, this is sufficient and vital for the induction of vasculogenesis [44]. Inappropriate production of VEGF by inflammatory cells might lead to dysfunctional vascularization, which affects the healing process [45]. In the lung, we show that about 46% of infiltrated SSC^low^CD11b^+^Ly6C^+^ monocytes are positive for VEGF at the baseline, while 82% of them are VEGF-positive at 6 hr post-exposure (Fig 3D). During the resolution stage, these recruited Ly6C^+high^ monocytes undergo Fas-dependent apoptosis [46]. This may help return intrapulmonary VEGF to pre-injury levels, and lung epithelial cells become the primary source of VEGF production. Studies have suggested that VEGF may serve as an alveolar epithelial cell growth factor, indicating its protective role in alveolar space [18]. Moreover, a study showed high-stretch ventilation induces early fibroproliferative responses with marked increases in lung tissue myofibroblast counts, collagen contents and BALF TGF-β. However, BALF keratinocyte growth factor (KGF) was significantly increased starting at 14 days [47]. The early fibroproliferative responses may be secondary to the alveolar epithelial cell damage incurred by high-stretch ventilation, and thus timely repair of alveolar epithelium is crucial for the resolution of VILI. Studies have shown that HGF exerts the most growth-promoting activity for type II cells, in comparison with EGF, tumor growth factor-α, acidic fibroblast growth factor, and KGF [48, 49]. If KGF increases at the late stage of injury repair [47], HGF and other growth-promoting factors may merit further investigation to enhance our understanding of their role in injury repair and the development of related therapeutic strategies.

In conclusion, the current study established a relevant experimental model to reflect the clinical situations in which patients acquire infections and, at the same time, require ventilator support. We provide evidence that pulmonary infiltrated Ly6C^+high^ monocytes are crucial for the development of VILI. The Ly6C^+high^ monocyte-VEGF functional axis detailed in this work provides mechanistic insights for preventing and/or treating clinical VILI.
